# Stability of small ubiquitin-like modifier (SUMO) proteases OVERLY TOLERANT TO SALT1 and -2 modulates salicylic acid signalling and SUMO1/2 conjugation in *Arabidopsis thaliana*

**DOI:** 10.1093/jxb/erv468

**Published:** 2015-10-22

**Authors:** Mark Bailey, Anjil Srivastava, Lucio Conti, Stuart Nelis, Cunjin Zhang, Hannah Florance, Andrew Love, Joel Milner, Richard Napier, Murray Grant, Ari Sadanandom

**Affiliations:** ^1^Biological & Biomedical Sciences, Durham University, Durham DH1 3LE, UK; ^2^ Present address: Plant proteolysis and signalling laboratory, School of Biosciences, University of Birmingham, Edgbaston, Birmingham B15 2TT, UK; ^3^Department of BioSciences, Università degli Studi di Milano, Via Celoria 26, 20133 Milano, Italy; ^4^Geoffrey Pope Building, Biosciences, College of Life and Environmental Sciences, University of Exeter, Stocker Road, Exeter EX4 4QD, UK; ^5^Cell and Molecular Sciences, The James Hutton Institute, Dundee DD2 5DA, UK; ^6^Plant Science Group, School of Life Sciences, College of Medical Veterinary & Life Sciences, University of Glasgow, Glasgow G12 8QQ, UK; ^7^School of Life Sciences, University of Warwick, Gibbet Hill Road, Coventry CV4 7ES, UK

**Keywords:** *Arabidopsis thaliana*, defence, pathogen, salicylic acid (SA), small ubiquitin-like modifier (SUMO), SUMO protease, SUMOylation.

## Abstract

Small ubiquitin-like modifier proteases 1 and 2 (SUMO1/2) have been linked to the regulation of salicylic acid (SA)-mediated defence signalling in *Arabidopsis thaliana*. In order to define the role of the SUMO proteases OVERLY TOLERANT TO SALT1 and -2 (OTS1/2) in defence and to provide insight into SUMO1/2-mediated regulation of SA signalling, we examined the status of SA-mediated defences in *ots1/2* mutants. The *ots1 ots2* double mutant displayed enhanced resistance to virulent *Pseudomonas syringae* and higher levels of SA compared with wild-type (WT) plants. Furthermore, *ots1 ots2* mutants exhibited upregulated expression of the SA biosynthesis gene *ICS1* in addition to enhanced SA-responsive *ICS1* expression beyond that of WT. SA stimulated OTS1/2 degradation and promoted accumulation of SUMO1/2 conjugates. These results indicate that OTS1 and -2 act in a feedback loop in SA signalling and that *de novo* OTS1/2 synthesis works antagonistically to SA-promoted degradation, adjusting the abundance of OTS1/2 to moderate SA signalling. Accumulation of SUMO1/2 conjugates coincides with SA-promoted OTS degradation and may play a positive role in SA-mediated signalling in addition to its repressive roles reported elsewhere.

## Introduction

The small ubiquitin-like modifier (SUMO) takes its name from its similarity to the well-studied post-translational modifier ubiquitin and is conserved throughout all kingdoms of eukaryotes (Müller *et al.*, 2001). In plants, covalent attachment of SUMO (SUMOylation) has been implicated in most life processes with a principal role in stress responses. Exposure to abiotic stresses such as heat shock and high salt concentrations leads to accumulation of SUMO conjugates (Kurepa *et al.*, 2003; Lois *et al.*, 2003; Conti *et al.*, 2008; Miura and Hasegawa, 2010).

Synthesized as an inactive precursor, SUMO proteins are processed to their mature form by SUMO proteases that cleave the C-terminal tail from the precursor exposing a diglycine motif—the site at which SUMO is attached to permissible lysine residues in substrate proteins. In plants, covalent conjugation of SUMO occurs most frequently on proteins containing a somewhat conserved motif, ψ-K-V-D/E (where ψ=any hydrophobic residue), facilitated by the sequential activity of three enzymes (E1, E2, and E3). In *Arabidopsis*, the E1 SUMO-activating enzymes AtSAE1 and AtSAE2 act as a heterodimer, responsible for adenylation-mediated ATP-dependent thiol-ester bond formation between SAE2 and SUMO. Transesterification results in the transfer of SUMO to the E2 SUMO-conjugating enzyme AtSCE1. AtSCE1 finally catalyses SUMO isopeptide bond formation to target proteins, in conjunction with E3 SUMO ligases HIGH PLOIDY 2 (AtHPY2/AtMMS21) or SAP and MIZ1 (AtSIZ1) (Miura *et al.*, 2005, 2007; Saracco *et al.*, 2007; Huang *et al.*, 2009; Ishida *et al.*, 2012; Novatchkova *et al.*, 2012). Once covalently bound, SUMO can alter a conjugated protein’s stability and/or functionality. SUMO may facilitate new protein–protein interactions through SUMO-interacting motifs (SIMs), and compete with other post-translational modifications such as ubiquitination and acetylation (Müller *et al.*, 2001; Kerscher, 2007). In addition to their SUMO processing activities, SUMO proteases also possess deconjugative activity capable of cleaving SUMO from target proteins, providing reversibility and buffering to the pathway (Mukhopadhyay and Dasso, 2007; Hickey *et al.*, 2012).

Salicylic acid (SA) is a plant hormone with a central role in mounting effective defences during pathogen challenge, both locally and systemically, in addition to being implicated in the regulation of growth and development (Vlot *et al.*, 2009). Plants produce SA from chorismic acid through two biosynthetic pathways, one catalysed by the PHENYLALANINE LYASE1 to -4 ( (AtPAL1–4) and the other by ISOCHORISMATE SYNTHASE1 and -2 (AtICS1 and -2) (Dempsey *et al.*, 2011). *atics1* mutants (also known as *salicylic acid induction-deficient2* or *sid2*) are defective in pathogen-induced SA biosynthesis and deficient in defence signalling, ICS1 being required for 90–95% of SA produced under avirulent *Pseudomonas syringae* challenge (Wildermuth *et al.*, 2001).

The discovery of SA binding capacity in NON-EXPRESSOR OF PATHOGENESIS-RELATED GENE1 (NPR1), and of its homologues NPR3 and NPR4, has led to the belief that, collectively, they are canonical SA receptors (Liu *et al.*, 2004; Zhang *et al.*, 2006; Attaran and He, 2012; Fu *et al.*, 2012; Wu *et al.*, 2012). NPR1, through interaction with members of the TGA family of bZIP transcription factors, co-activates SA-mediated defence (Cao et al., 1994, 1997; Dong, 2004; Kesarwani *et al.*, 2007; Mukhtar *et al.*, 2009). NPR3 and NPR4 participate as subunits in cullin RING ubiquitin E3 ligase-mediated ubiquitination. Differential affinity of NPR3 and NPR4 for SA and opposing mediation of SA binding upon their interaction with NPR1 have provided a molecular mechanism for sensing SA levels in the cell and activating defence responses appropriately (Fu *et al.*, 2012; Kawano and Bouteau, 2013).

The role of SUMO1/2 in the regulation of SA biosynthesis and defence against *Pseudomonas syringae* pv. *tomato* (*Pst*) has emerged from mutation of the SUMO E3 ligase SIZ1 and was further substantiated with knockdown of either SUMO1 or -2 in the mutant background of the other (SUMO1 or -2), which led to increases in SA, SA-*O*-β-glucoside (SAG), and *Pst* resistance (Lee *et al.*, 2006; van den Burg *et al.*, 2010). These findings have indicated that SUMO1/2 suppress activation of SA-mediated responses via the SIZ1 SUMO ligase (van den Burg and Takken, 2010).

Previously, we showed that overexpression of SUMO proteases OVERLY TOLERANT TO SALT1 and -2 (OTS1 and -2) promotes salt stress tolerance and that degradation of OTS1 and -2 is induced by salt (Conti *et al.*, 2008). The *ots1 ots2* double mutant was shown to accumulate SUMO1/2 conjugates. Given the associations made between SUMO1/2, SA-mediated defences, and between SA and salt stress tolerance (reviewed by Horváth *et al.*, 2007; Miura and Tada, 2014), we decided to investigate the role of SUMO proteases OTS1 and -2 in defence, and provide further insight into the regulation between the SUMO system and SA signalling. We showed that OTS1 and -2 negatively regulate SA biosynthesis and propose that *de novo* synthesis and SA-promoted degradation of OTS1/2 antagonistically adjust the abundance of this negative regulator depending on the level of pathogen threat. Furthermore, we provide evidence that accumulation of SUMO conjugates results from SA-promoted degradation of OTS1/2 and may play a signalling role.

## Materials and methods

### Plant growth conditions

*Arabidopsis thaliana* was grown in Panasonic MLR plant growth chambers with a day/night cycle of 10h in the light at 22 °C and 14h of dark at 20 °C with a constant relative humidity of 70% on Levington F2 compost plus sand. The *ots1* (At1g60220) and *ots2* (At1g10570) null-mutant lines were isolated from T-DNA insertion lines SALK 022798 and SALK 001579, respectively, as described previously (Conti *et al.*, 2008).

### *P. syringae* infection assays

*Pst* DC3000 was grown on King’s B agar with 50 μg ml^–1^ of rifamycin and incubated for 2 d at 28 °C. Liquid King’s B medium supplemented with rifamycin was inoculated from plates and grown at 28 °C overnight shaking at 200rpm. Cells were centrifuged at 5000*g* at room temperature and resuspended in sterile water; this was repeated once and the final suspension was diluted to an optical density at 600nm (OD_600_) of 0.002 (1×10^6^ colony-forming units ml^–1^). Five leaves each from 12 4-week-old plants were pressure infiltrated with the suspension and returned to the growth chamber. Leaf discs were cut from three random leaves and macerated in 200 μl of sterile water and serially diluted 1:5 in a multiwell plate. A volume of 15 μl of each dilution was spotted onto Kings B/rifamycin agar plates and allowed to dry. The plates were incubated for 24h before counting the colonies. This was repeated three times per genotype per day (Katagiri *et al.*, 2002).

### Trypan blue staining

Visualization of dead cells using trypan blue staining was performed on 4-week-old *Arabidopsis* plants pressure infiltrated with *Pst* DC3000 (as described above) with a final bacterial suspension OD_600_ of 0.2 (1×10^8^ colony-forming units ml^–1^). Three leaves were detached per genotype per time point (untreated and 6, 12, 24, and 36h post infiltration). These were then boiled in trypan blue staining solution (Ma *et al.*, 2011) for 10min and left in the stain overnight. The stain was then poured off and replaced with destaining solution (saturated chloral hydrate). Samples were inverted and left for 4h. The destain was changed four times before leaves were imaged. Triplicate leaves were photographed using a Nikon D30 with a macro lens on a light box. Individual leaf images were taken using an Olympus SZH10 research stereo dissecting light microscope and QImaging QICAM camera. Images of leaves at a magnification of ×10 were taken using a Zeiss Axioskop light microscope and QImaging RETIGA 2000R camera. The percentage of cell death was quantified using triplicate leaves using the ‘Analyze particles’ function of ImageJ (version 1.47).

### SA and MG132 treatments

SA (Sigma-Aldrich; 400mM stock in ethanol) was diluted in sterile water to a final concentration of 2mM with the addition of 0.005% Silwett L70 for spray treatments. Equal volumes were sprayed (*Arabidopsis*) or infiltrated (*Nicotiana benthamiana*). Control treatments were carried out with equivalent volumes of ethanol and Silwett. The plants were sealed using propagator lids and returned to the growth chambers. Aerial tissues (*Arabidopsis*) or leaves (*N. benthamiana*) from three plants were removed at each time point and frozen in liquid nitrogen for RNA or protein extraction (see below). SA and MG132 treatments were performed on 10-d-old transgenic *Arabidopsis* seedlings grown in liquid ½ Murashige and Skoog medium (OTS1) or on 4-week-old *N. benthamiana* transiently expressing OTS2 (see below). MG132 treatments were performed at a final concentration of 20 μm (*Arabidopsis*) or 50 μm (*N. benthamiana*) (stock 10mM dissolved in DMSO) or with solvent (control) for 1h. *Arabidopsis* was treated with a final concentration of 0.5mM SA or an equivalent volume of ethanol mixed into the medium and incubated for a further 30min. *N. benthamiana* leaves were infiltrated with a final concentration of 2mM SA simultaneously with MG132 and incubated for 1h. Plant tissues were immediately frozen in liquid nitrogen for protein extraction.

### RNA extraction, cDNA synthesis, and quantitative PCR (qPCR)

Leaf tissue frozen in liquid nitrogen was ground to a fine powder in a pre-chilled pestle and mortar. A Spectrum^TM^ Plant Total RNA kit (Sigma-Aldrich) was used to extract RNA following the manufacturer’s recommendations. The RNA was quantified by measuring the absorbance at wavelengths of 260 and 280nm using a NanoDrop^TM^ 1000 Spectrophotometer (Thermo Scientific). The RNA was DNase treated with Promega DNase I following manufacturer’s guidelines. cDNA synthesis was undertaken with Invitrogen SuperScript^®^ II Reverse Transcriptase following manufacturer’s guidelines. The RNA was tested for the absence of contaminating genomic DNA by PCR using a primer spanning an exon junction. Quantitative PCR primers were designed to gene targets using the National Center for Biotechnology Information Primer-BLAST, and primer annealing was tested using gradient PCR. Relative expression was compared between genotypes using target primers and primers to the housekeeping gene *ACTIN7* (At5g09810) for normalization. SYBR^®^ Green JumpStart^TM^ Taq ReadyMix^TM^ (Sigma-Aldrich) was used in conjunction with Rotor- Gene^®^ Q (Qiagen) and analysis was undertaken with the software provided using the comparative quantification method (Warton *et al.*, 2004). Graphs and statistical analysis were produced using GraphPad Prism version 6.0 for Mac (GraphPad Software, http://www.graphpad.com).

### Protein extraction, quantification, and Western blotting

Frozen plant tissue was ground to a fine powder with a chilled pestle and mortar. *Arabidopsis* extraction buffer (50mM Tris/HCl, pH 8.5, 4% SDS, 2% β-mercaptoethanol, 10mM EDTA) or *N. benthamiana* extraction buffer (ground with polyvinylpolypyrrolidone; 5mM Tris/HCl, pH 7.5, 150mM NaCl, 1 μM EDTA, 10% glycerol, 0.1% Triton X-100 and 10mM dithiothreitol with protease inhibitor tablet) was added 1:1 w/vol. The mixture was centrifuged at 12 000*g* at 2 °C for 10min. The protein concentration was determined using a Direct Detect^TM^ Infra-red Spectrometer (EMD Millipore) and samples were equalized with the addition of extraction buffer. Laemmli sample buffer (4×) was added and the samples were separated on 12/15% polyacrylamide gels. The proteins were transferred to a polyvinylidene difluoride (PVDF) membrane overnight. The blotted membranes were blocked with 5% semi-skimmed milk powder for 1h at room temperature and probed with the following antibodies: rabbit anti-SUMO1 polyclonal antibody (Abcam) and anti-HA RAT monoclonal antibody (3F10; Roche) both used at 1:5000 and 1:10 000 dilutions in TBST (Tris/HCl, pH 7.5, with 150mM NaCl ansd 0.1% Tween 20), for 4 and 3h, respectively. Secondary horseradish peroxidase (HRP)-conjugated anti-rabbit and anti-rat antibodies (Sigma-Aldrich) were applied at 1:20 000 for 1h before developing the blots with X-ray film using an automated developer.

### *Agrobacterium*-mediated transient expression

*Agrobacterium tumefaciens* strain GV3101::pmp90 was transformed with OTS2 pEG201 and grown in liquid Luria–Bertani medium supplemented with rifamycin, gentamycin, and kanamycin overnight with shaking (200rpm) at 28 °C. The cultures were adjusted to an OD_600_ of 0.2 and infiltrated into the leaves of 4-week-old *N. benthamiana* plants. The plants were incubated at room temperature for 3 d prior to SA and MG132 treatments (see above). The proteins were extracted and analysed by western blotting as above.

### SA measurement

Freeze-dried leaf powder (10mg) was extracted in 0.8ml of 80% methanol containing a 100 µM internal standard. After centrifugation (10min at 16 100*g*, 4 °C), the samples were filtered through a 0.2 μm (PVDF) syringe filter (Chromacol). Hormone quantitative analysis was performed using an 6420B triple quadrupole mass spectrometer (Agilent Technologies) joined to a 1200 Series Rapid Resolution HPLC system (Agilent Technologies). Sample extract (20 µl) was loaded onto a Zorbax Eclipse Plus C18 3.5 µm, 2.1×150mm reverse-phase analytical column (Agilent Technologies). The following gradient was used: 0min, 0% B; 1min, 0% B; 5min, 20% B; 20min, 100% B; 25min, 100% B; 27min, 0% B; 7min post time. The triple quadrupole source conditions were as follows: gas temperature 350 °C, drying gas flow rate 9 l min^–1^, nebulizer pressure 35 psig, capillary voltage ±4kV. The fragmentor voltage and collision energies were optimized for each compound (Pan *et al.*, 2010).

## Results

### The *ots* double mutant displays enhanced resistance to virulent *P. syringae* and constitutively active defences

**Fig. 1.**
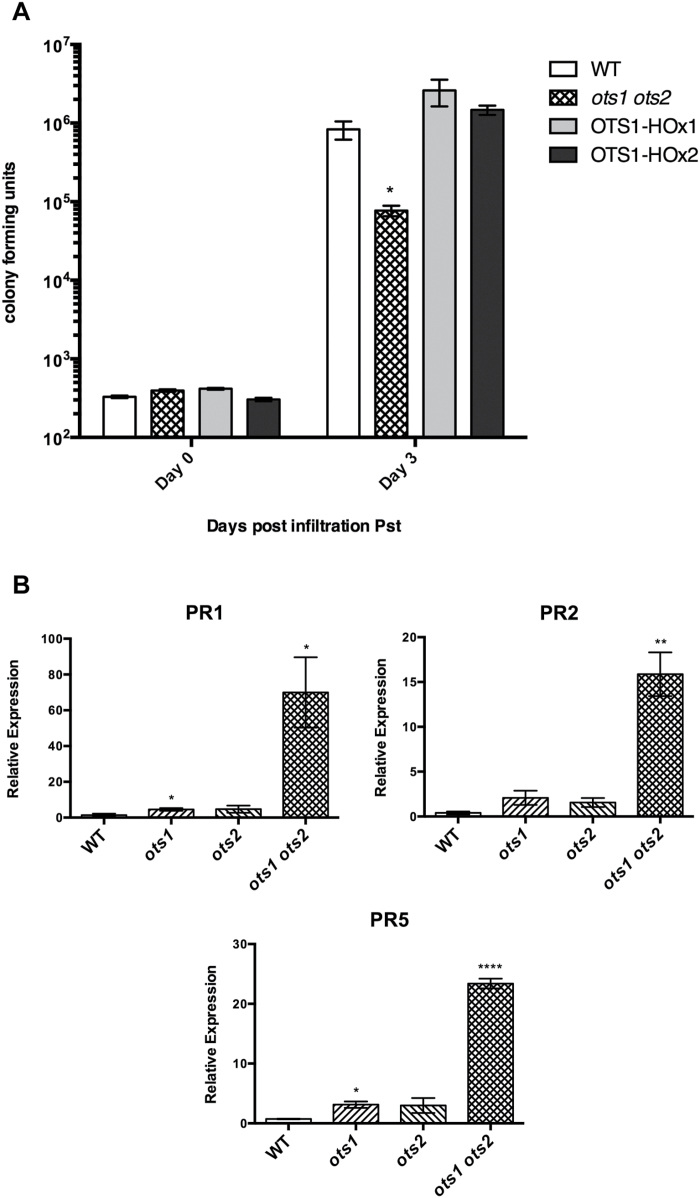
The *ots1 ots2* double mutant displays enhanced resistance to virulent *Pst.* (A) Colony-forming unit counts of *Pst* DC3000 in the leaves of 4-week-old WT, *ots1 ots2* double mutant, and OTS1-overexpressing lines (OTS1-HOx1 and OTS-HOx2) on the day of infiltration (day 0) and on day 3. (B) Quantitative PCR analysis of gene expression from 4-week-old WT, single *ots1* and *ots2* mutants, and the double *ots1 ots2* mutant of *PATHOGENESIS-RELATED1* (*PR1*), *PR2*, and *PR5* genes (normalized to *ACTIN7*). Error bars represent SEM. *P* values for differences between WT and mutants: *P<0.05, **P<0.01, and ****P<0.0001 (one-way ANOVA with Tukey post hoc test ).(one-way ANOVA with Tukey post hoc test).

**Fig. 2.**
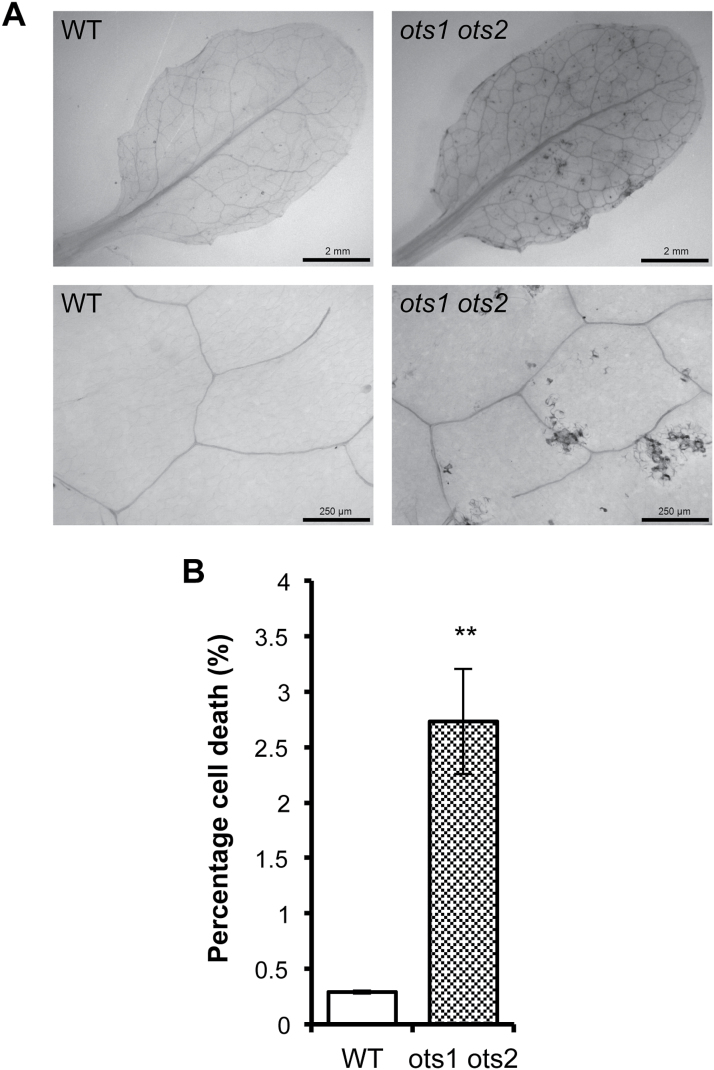
The *ots1 ots2* double mutant displays spontaneous lesions. Trypan blue staining for cell death within comparable leaves from 2-week-old WT and *ots1 ots2* double mutant plants. (A) Representative images of stained leaves. (B) Analysis of the percentage of cell death per 4mm^2^ across biological replicates using ImageJ. Error bars represent SEM. *P* values for differences between WT and mutants: **P=0.001–0.01 (unpaired Student’s *t*-test).

Mutants of the SUMO E3 ligase SIZ1 display reduced levels of SUMO conjugates and enhanced resistance to virulent pathogens relative to WT plants (Lee *et al.*, 2006). We have shown previously that the *ots1 ots2* double SUMO protease mutant accumulates higher levels of SUMO conjugates than WT plants (Conti *et al.*, 2008, 2009). Therefore, we decided to investigate the status of defence responses in the SUMO protease mutants. Surprisingly, growth of the virulent bacterial plant pathogen *Pst* was 10 times lower in the *ots1 ots2* double mutant compared with WT Columbia-0 plants (Fig. 1A), while WT plants transformed with constructs overexpressing *OTS1* driven by the cauliflower mosaic virus 35S promoter (OTS1-HOx1 and -2) did not exhibit significantly different susceptibility to virulent *Pst* compared with non-transformants (Fig. 1A). The single T-DNA insertion mutants *ots1* and *ots2* did not differ significantly in susceptibility compared with WT plants (see Supplementary Fig. 1 at *JXB* online), indicating that OTS1 and OTS2 may act redundantly in defence suppression. In order to understand more about the defence phenotype of the *ots* mutants, basal gene expression of the pathogenesis-related (PR) defence genes *PR1*, *PR2*, and *PR5* were measured by qPCR. Transcript levels (normalized to the housekeeping gene *ACTIN7*) were significantly higher in the *ots1 ots2* double mutant compared with WT (Fig. 1B). Trypan blue staining of untreated leaves to visualize cell death revealed that the *ots1 ots2* double mutants had developed spontaneous lesions of dead cells that were absent in WT plants (Fig. 2A). Image analysis further confirmed these lesions to be significantly more prevalent in the *ots1 ots2* double mutant (Fig. 2B). Taken together, these results suggested that OTS1 and OTS2 work redundantly to restrict processes culminating in cell death, but by doing so they compromise defence against virulent *Pst* in *Arabidopsis*.

### SA signalling and biosynthesis is upregulated in the *ots* double mutant

**Fig. 3.**
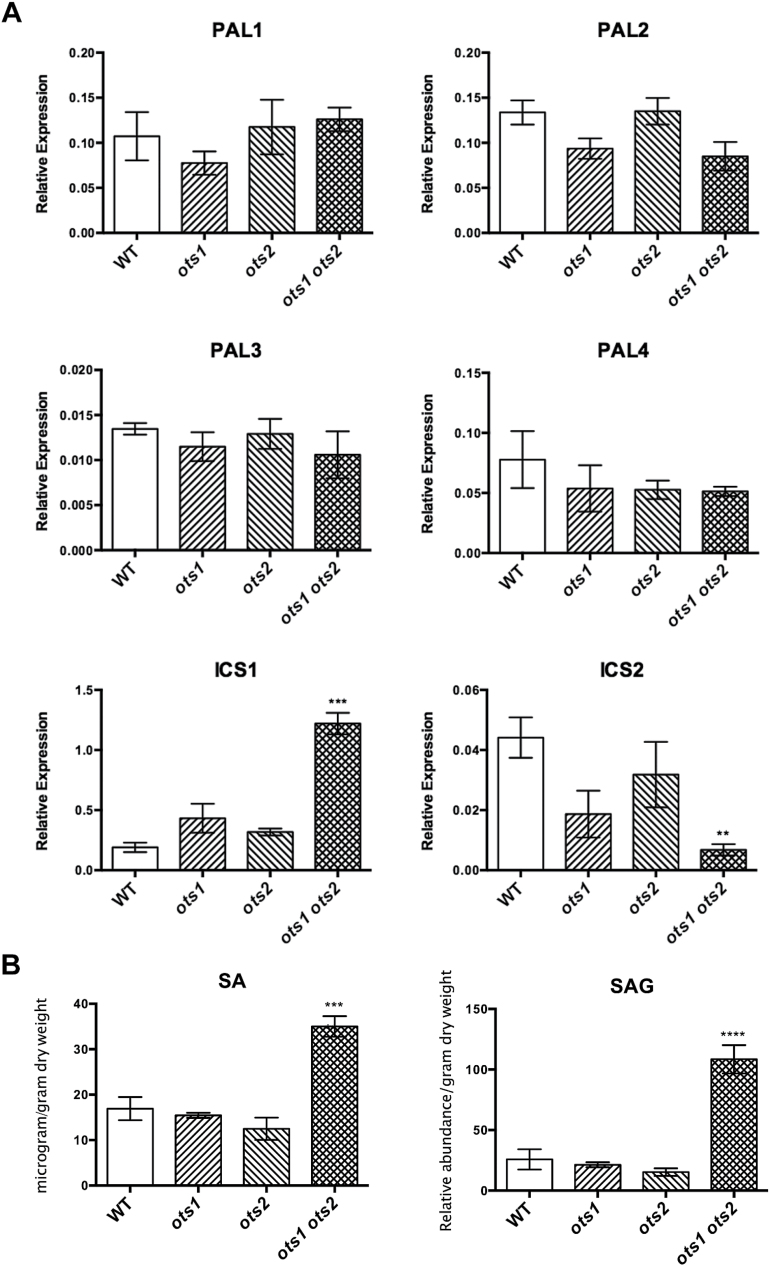
SA biosynthesis is upregulated in the *ots1 ots2* double mutant. (A) qPCR analysis of gene expression from 4-week-old WT, single *ots1* and *ots2* mutants, and the double *ots1 ots2* mutant, of SA biosynthesis genes *ICS1* and *PAL1 to -4* (normalized to *ACTIN7*). (B) Liquid chromatography/mass spectrometry quantification of SA and glycosylated SA (SAG) in WT, single *ots1* and *ots2* mutants, and the double *ots1 ots2* mutant. Internal standards were unavailable for SAG; hence, values are given as relative abundances (one-way ANOVA with Tukey’s post hoc test). Error bars represent SEM. *P* values for differences between WT and mutants: **P<0.01, ***P<0.001 and ****P<0.0001, respectively (one-way ANOVA with Tukey’s post hoc test).

In order to ascertain whether increased SA biosynthesis underlies the enhanced resistance and cell death, we examined the expression of the genes encoding SA biosynthetic enzymes. In the *ots1 ots2* double mutants, *PAL1* to *-4* transcript levels were similar to WT, whereas *ICS1* and -*2* transcripts differed significantly (Fig. 3A). *ICS1* was upregulated over 6-fold, whereas *ICS2* was downregulated 6-fold relative to WT gene expression levels. The opposing regulation of *ICS1* and *ICS2* biosynthesis enzymes prompted us to measure SA concentrations *in planta* using liquid chromatography/mass spectrometry. SA and SAG concentrations were significantly higher in the *ots1 ots2* double mutant (Fig. 3B) than in the WT and *ots* single mutants. Upregulation of *ICS1* appears to lead to increased SA levels, consistent with previous findings showing that *ICS1* is responsible for the majority of pathogen-induced SA synthesis (Wildermuth *et al.*, 2001). Furthermore, the recent finding that the dwarf phenotype of *EARLY FLOWERING SHORT DAY4* (*ESD4*) SUMO protease mutants can be partially recovered by mutation of *ICS1* supports this conclusion (Villajuana-Bonequi *et al.* 2014). Thus, these results highlight the importance of SUMO proteases in regulating SA biosynthesis and signalling.

**Fig. 4.**
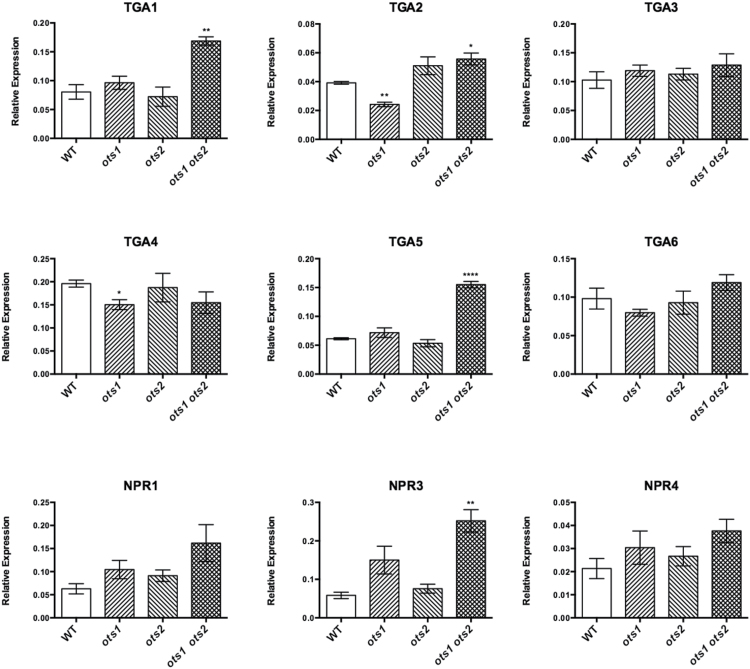
SA-related defence gene expression is upregulated in the *ots1 ots2* double mutant. qPCR analysis of gene expression from 4-week-old WT, single *ots1* and *ots2* mutants, and the double *ots1 ots2* mutant, of SA signalling pathway components, the TGA transcription factors *TGA1*, *-2*, *-3*, *-4*, *-5*, and *-6* and *NPR1* and its paralogues *NPR1-LIKE PROTEIN3* (*NPR3*) and *NPR4* (normalized to *ACTIN7*). Error bars represent SEM. *P* values for differences between WT and mutants: *P=0.01–0.05, **P=0.001–0.01, and ****P< 0.0001, respectively (one-way ANOVA with Tukey’s post hoc test).

In order to identify potential perturbations in the SA pathway, basal expression of key components of the molecular signalling pathway was determined using qPCR. Transcripts of the bZIP TGA transcription factors *TGA1*, *TGA2*, and *TGA5* were significantly higher in the *ots1 ots2* double mutant relative to WT (Fig. 4). Increased levels of transcripts of the SA receptors were also found, in particular *NPR3*, which, with its proposed role in cell death promotion at high SA concentrations, may be facilitating the spontaneous lesion phenotype of the *ots1 ots2* double mutant (Fu *et al.*, 2012).

### OTS1 and -2 limit SA biosynthesis through restriction of *ICS1* gene expression

**Fig. 5.**
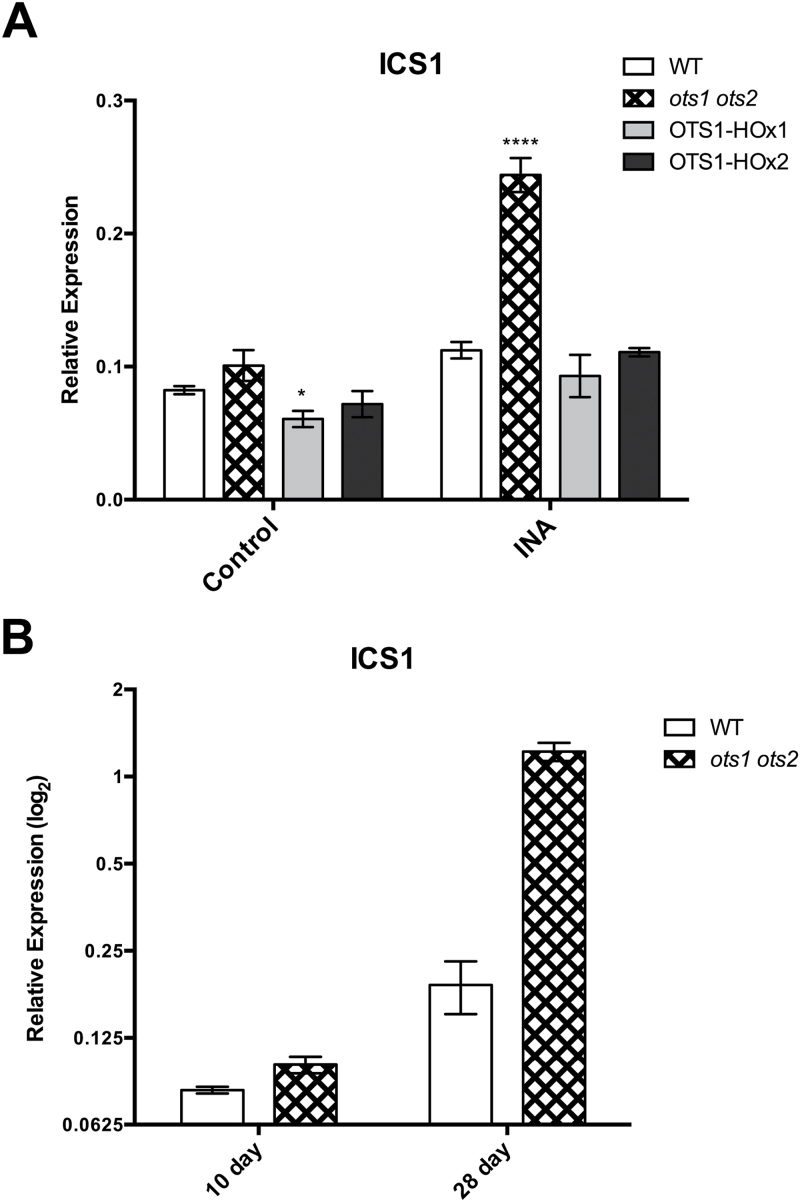
OTS1 and -2 restrict *ICS1* gene expression. qPCR gene expression analysis of *ICS1* in (A) 10-d-old WT, *ots1 ots2* double mutant and transgenic *OTS1*-overexpressing (OTS1-HOx1 and -2) plants grown in the presence of INA (40mg ml^–1^) or solvent (control) (A) and in 10- and 28-d-old WT and the *ots1 ots2* double mutant (B). Error bars represent SEM. *P* values for differences between WT and mutants: *P=0.01–0.05, ****P<0.0001, respectively (multi-way ANOVA with Tukey test post hoc).

*ICS1* gene expression is positively regulated by SA (Hunter *et al.* 2013), which was further demonstrated here in response to the SA functional analogue 2,6-dichloropyridine-4-carboxylic acid (INA) (see Supplementary Fig. 3 at *JXB* online) (Conrath *et al.*, 1995). As the *ots1 ots2* double mutants accumulated SA (Fig. 3B) and possessed elevated *ICS1* transcription (Fig 3A), we examined the status of this positive feedback in the *ots1 ots2* double mutant and in OTS1 overexpressors. Ten-day-old seedlings were grown in the presence or absence of 40 μm INA. Interestingly, the double *ots1 ots2* mutant showed a significantly greater capacity for induction of *ICS1* under INA (Fig. 5A, multi-way ANOVA with Tukey’s post hoc test). This suggests that the mutants lack the restrictive regulation of *ICS1* gene transcription present in WT plants, which only displayed a low-level responsiveness to INA. Furthermore, the OTS1-overexpressing line OTS1-HOx1 exhibited significantly lower *ICS1* expression in untreated seedlings, demonstrating the ability of OTS1 SUMO protease to repress *ICS1* expression (Fig. 5A). These results demonstrated that OTS1 and -2 provide negative feedback in SA signalling, presumably to prevent inappropriate activation of defences. Furthermore, the results in Fig. 5A suggest that OTS1/2 may antagonize SA-mediated defence during pathogen challenge to adjust the response to suitable levels.

Previously, a significant difference between the *ots1 ots2* double mutant and WT *ICS1* transcript abundance was observed in mature plants (Fig. 3A). It was noticeable that in 10-d-old seedlings expression of *ICS1* was lower and that the differences between WT and the *ots1 ots2* double mutant were much smaller (Fig. 5A). Comparison of *ICS1* gene expression between genotypes in 10- and 28-d-old plants indicated that *ICS1* transcript abundance increases as plants mature (Fig. 5B). Similar to INA treatment, *ICS1* expression appeared to be less restricted in the *ots1 ots*2 double mutant as the plants developed. Thus, OTS1 and -2 appear to play a role in restricting the SA pathway during plant development in addition to SA-mediated immunity.

### SA promotes degradation of the SUMO proteases OTS1/-2 and SUMO1/2 conjugation

Given the evidence presented here that the OTS SUMO proteases negatively regulate SA signalling (Figs 1–5), one would expect OTS1 and -2 activities to be downregulated during SA-mediated defence activation in WT plants. *OTS1* and *-2* gene expression did not alter in response to treatment with SA or the SA functional analogue INA (see Supplementary Figs 2A, B and 3 at *JXB* online) (Conrath *et al.*, 1995). This may indicate that SA facilitates regulation of the OTS proteases post-translationally and led us to examine the effects of SA upon OTS1/2 stability.

**Fig. 6.**
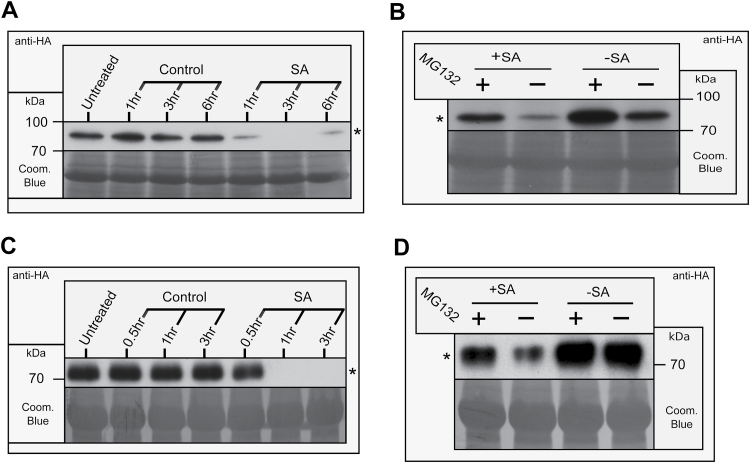
OTS1 and -2 degradation are promoted by SA. Western blots probed with anti-HA monoclonal antibodies showing OTS1 and -2 stability using the *Arabidopsis* transgenic line OTS1-HOx2 (A, B) or *Agrobacterium*-mediated transient expression in *N. benthamiana* of OTS2 (C, D). (A) OTS1: 4-week-old plants sprayed with SA or solvent (control) over a 6h time course. (B) OTS1: 10-d-old plants, grown in liquid ½ MS treated with SA (+SA) or solvent (–SA) following pre-incubation with the 26S proteasome inhibitor MG132 (+) or solvent (−). (C) OTS2: 4-week-old plants infiltrated with SA or solvent (control) over a 3h time course. (D) OTS2: 4-week-old plants infiltrated with SA (+SA) or solvent (–SA) following pre-incubation with MG132 (+) or solvent (−). Asterisks (*) indicate the HA-OTS1/2 bands. Coomassie blue (Coom. Blue) staining of the blots is shown as a loading control.

The OTS1-overexpressing line OTS1-HOx2 expressing an N-terminal fusion to a human influenza hemagglutinin (HA) epitope tag was used to monitor OTS1 protein stability. We previously established that N-terminal fusions to the OTS proteins do not impede protease activity (Conti *et al.*, 2008). Plants sprayed with SA showed a depletion of OTS1 after 1h, with no OTS1 visible at 3h after spraying (Fig. 6A). Seedlings treated with SA for 30min, with pre-incubation with the 26S proteasomal inhibitor MG132, showed reduced depletion of OTS1 relative to control seedlings, suggesting a proteasomal route of degradation (Fig. 6B). The effect of SA on OTS2 stability was further examined by *Agrobacterium*-mediated transient expression in *N. bethamiana* of OTS2 N-terminally fused to HA. Depletion of the OTS2 protein was similarly observed 1h after SA treatment and appeared to be dependent on proteasome function (Fig. 6C, D). Clearly, OTS SUMO protease abundance is SA dependent, when taken together with previous results that OTS1/2 function as negative regulators of SA signalling (Figs 4 and 5). This highlights SUMO protease stability as a key mechanism of SA signalling regulation.

**Fig. 7.**
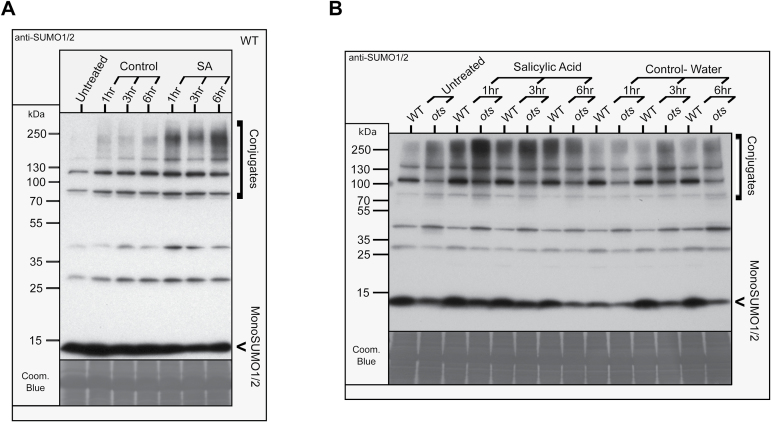
SUMO1/2 conjugation is promoted by SA. Western blots probed with anti-SUMO1/2 polyclonal antibodies showing SUMO1/2 and their conjugates in 4-week-old plants sprayed with salicSAylic acid or solvent (control). (A) Effect on WT over 6h. (B) Effect on WT compared with the *ots1 ots2* double mutants over 6h. Coomassie blue staining (Coom. Blue) of the blots is shown as a loading control.

Next, we decided to examine the effects of SA, in terms of OTS1/2 degradation, on SUMO conjugation *in planta*. WT plants showed enhanced accumulation of SUMO1/2 conjugates within 1h of exogenous application of SA, coinciding with the OTS1/2 degradation seen previously (Figs 6A, C and 7A). SUMO conjugate levels appeared to decrease slightly at 3h after SA treatment, although the levels appeared to peak at 6h post-treatment. In the *ots1 ots2* double mutant, SUMOylated proteins also accumulated within 1h of SA treatment, and their levels remained elevated for up to 6h, indicating that other SUMO proteases present in *Arabidopsis* are sufficient to produce mature conjugatable SUMO1/2 (Fig. 7B). Significantly, compared with WT, increased levels of high-molecular-weight (~250kDa) conjugates accumulated in SA-treated *ots1 ots2* double mutant plants 1h after treatment, indicative of hyperaccumulation of polySUMOylated conjugates. As SA promotes OTS1 and -2 degradation (Fig. 6), this suggests that *de novo* OTS1/2 protein synthesis is dampening SUMO conjugate accumulation in WT plants. The levels of SUMO1/2 monomers (~11kDa) also appear ed to be reduced in the *ots1 ots2* double mutants (Fig. 7B). This was probably due to reduced recycling of conjugates from deSUMOylation, as although SA appears to induce more SUMO conjugation, there was no evidence for additional production of SUMO or processed SUMO in the double mutant lines. Reports elsewhere indicating that *SUMO1* and *SUMO2* gene expression are unresponsive to SA support this idea (van den Burg *et al.*, 2010); we confirmed this finding in plants grown on plates in the presence of the SA analogue INA (see Supplementary Fig. 4 at *JXB* online). These results indicate that SUMO conjugation seen in response to SA treatment is likely to be the result of SA-promoted SUMO protease degradation and may play a signalling role in SA-responsive molecular pathways.

Collectively, the results presented here indicate that OTS1/2 SUMO proteases function as negative regulators of *ICS1* expression and hence SA production. Unrestricted SA signalling observed in the absence of the OTS proteins (*ots* mutants) (Figs 3A and 5A) together with the SA-promoted OTS degradation (Fig. 5) suggest that OTS1/2 stability is central to the regulation of SA signalling. We propose that the balance between *de novo* synthesis and SA-promoted degradation antagonistically adjusts the abundance of OTS1/2 depending on the level of pathogen threat, acting to prevent inappropriate escalation of immune responses and thus promote cell survival. We have also shown that elevated OTS1/2 degradation leads to the accumulation of SUMO1/2 conjugates, which may include modification of *ICS1* gene expression regulators and/or other signalling molecules in SA-responsive pathways.

## Discussion

Examination of the double SUMO protease mutant *ots1 ots2* revealed accumulation of SA and unrestricted expression of SA biosynthesis gene *ICS1* in this background. Taken with SA-dependent degradation of OTS1/2 proteases, we have highlighted the importance of OTS1/2 SUMO protease protein abundance in regulation of SA biosynthesis and immunity in *Arabidopsis*.

The differential affinity of SA receptors NPR3 and NPR4, and opposing mediation of SA binding upon their interaction with the NPR1 master SA regulator, have provided a molecular mechanism for sensing SA levels in the cell (Dong, 2004; Kawano and Bouteau, 2013; Fu *et al.*, 2012). We propose OTS1/2 SUMO proteases as negative regulators of SA signalling, adding another layer of regulation to this model through SA-dependent protein stability. In the absence of SA, OTS1/2 proteases prevent inappropriate activation of the SA pathway, restricting *ICS1* gene expression. Upon pathogen detection, SA biosynthesis is positively regulated and promotes degradation of OTS1/2, facilitating escalation of SA signalling, while *de novo* OTS1/2 synthesis provides negative feedback restricting *ICS1* gene expression presumably to prevent an excessive response to pathogen threat and facilitate recovery once a challenge has been overcome. Therefore, OTS1/2 act antagonistically to SA-mediated degradation of NPR1 via NPR3 promoting cell survival through restriction of ICS1-cataylsed SA synthesis and possibly of other signalling components such as NPR3 directly.

SUMO protease stability may regulate *ICS1* gene expression through its effects on SUMOylation. SA treatment led to SUMO conjugate accumulation simultaneously with OTS1/2 SUMO protease degradation. This poses SUMO protease degradation as a mechanism by which to shift the balance between SUMO conjugation and deconjugation towards conjugate accumulation, as observed in human cells (Xirodimas and Lane, 2008). (De-)SUMOylation of regulators of *ICS1* gene expression such as the repressors ETHYLENE INSENSITIVE3 (EIN3) and ETHYLENE INSENSITIVE3-LIKE1 (EIL1) or activators CAMODULIN-BINDING PROTEIN 60-LIKE G (CBP60G), SAR DEFICIENT 1 (SARD1), and WRKY28 (Chen *et al.*, 2009; van Verk *et al.*, 2011; Zhang *et al.*, 2010), may explain the impact of OTS1/2 protein stability on *ISC1* gene expression.

While the results presented here present strong evidence of the negative role of SUMO proteases in SA signalling, there remains controversy over the role of SUMOylation in SA regulation. Mutants of the SUMO E3 ligase SIZ1 exhibit significantly reduced levels of SUMO conjugation, while also accumulating greater levels of SA and showing constitutively activate pathogen defence responses (Lee *et al.*, 2006). A comparable phenotype was observed here in the double SUMO protease mutant *ots1 ots2*, and recently, in *esd4* SUMO protease mutants (Villajuana-Bonequi *et al.*, 2014). Both SUMO protease mutants possess higher levels of SUMO conjugates, indicating that previous conclusions that SUMO1/2 conjugation has a solely negative role in SA signalling appear to be incorrect (van den Burg and Takken, 2010). Previously, van den Burg *et al.* (2010) showed that overexpression or knockdown of *SUMO1* or *-2* leads to the accumulation of SA and SAG, in addition to activating SA-dependent defence responses, and concluded that the balance of unmodified and SUMOylated proteins appears to be important. Here, we have shown that SA treatments promote the degradation of OTS1/2 SUMO proteases and that this leads to SUMO1/2 conjugate accumulation, indicating the apparent existence of a feedback loop between SUMOylation and SA regulation. Overexpression of mutated SUMO1 and -2 lacking the attachment residues for conjugation was also reported to display heightened SA synthesis-related phenotypes (van den Burg *et al.*, 2010), indicating that accumulation of free SUMO monomers or SUMO bound to SUMOylation pathway enzymes may impact on SA biosynthesis, in addition to SUMO conjugation. This may be the case in *siz1* mutants, which lack the final enzyme to catalyse SUMO transfer from E2 to the substrate.

It is clear that we do not yet have the full picture of how SUMOylation and SA interact, and we are as yet unable to account for the consequences of SIMs on protein interactions. Defining how non-covalent SUMO-led interactions impact on this pathway and how the different SUMO homologues affect each other’s functioning will require further investigation. Nonetheless, we have shown that the SUMO proteases OTS1/2 play a clear role in negatively regulating SA signalling through *ICS1* expression restriction. Key questions remain: Are OTS deSUMOylating activities responsible for *ICS1* regulation or do they possess discrete functions? Are other defence-related genes regulated in a similar manner, such as NPR3? Are NPR1, -3 and -4 SA receptors responsible for inducing SA-promoted degradation of the OTS proteases? Once addressed these questions will help clarify the complex relationship between SUMOylation and SA biosynthesis.

We reported previously that overexpression of OTS1 leads to salt tolerance, and we found here that the *ots1 ots2* double mutant possessed elevated levels of SA (Conti *et al.*, 2008). Exogenous application of low concentrations of SA has been shown to alleviate abiotic stress-induced growth reduction, presumably due to enhancement of antioxidant enzyme expression and/ or altered ion channel fluxes, while high concentrations of SA cause oxidative damage resulting from hydrogen peroxide generation (Neuenschwander *et al.*, 1995; Aftab *et al.*, 2011; Jayakannan *et al.*, 2013; Fayez and Bazaid, 2014; Li *et al.*, 2014). Given that we have shown that SA promotes accumulation of SUMO1/2 conjugates and, previously, that SUMOylation of DELLAs mediates growth repression (Conti *et al.*, 2014), this would suggest that the levels of SA required to promote SUMOylation and arrest growth are higher than those that enhance abiotic stress-tolerant growth. Therefore, salt tolerance in the OTS overexpressors may be due to the restriction of SA to low levels (Shim *et al.*, 2003; Sawada *et al.*, 2007). The relationships between SA and salt tolerance and SUMO and SA biosynthesis highlight how crucial the abundance of either molecule is in terms of its signalling outcomes. Future studies need to go beyond the use of mutants and arbitrary overexpression to fully understand this challenging area of molecular signalling; the advancement of quantitative proteomics technologies may provide the answers.

## Supplementary data

Supplementary data are available at *JXB* online.

Supplementary Fig. 1. Colony-forming unit counts of *Pseudomonas syringae* pv. *tomato* DC3000 from the leaves of 4-week-old *Arabidopsis* plants.

Supplementary Fig. 2. *OTS1* and *OTS2* gene expression is unresponsive to SA treatment.

Supplementary Fig. 3. *OTS1* and *OTS2* gene expression is unresponsive to INA treatment.

Supplementary Fig. 4. *SUMO1* and *SUMO2* gene expression is unresponsive to INA treatment.

Supplementary Data
