# Supplementary material for: Stability of small ubiquitin-like modifier (SUMO) proteases OVERLY TOLERANT TO SALT1 and -2 modulates salicylic acid signalling and SUMO1/2 conjugation in Arabidopsis thaliana
Source: J Exp Bot. 2015 Oct 22;67(1):353–63. doi: 10.1093/jxb/erv468 (PMC4682439; doi:10.1093/jxb/erv468)
Supplement: Supplementary Data [file supp_67_1_353__index.html]

Stability of small ubiquitin-like modifier (SUMO) proteases OVERLY TOLERANT TO SALT1 and -2 modulates salicylic acid signalling and SUMO1/2 conjugation in Arabidopsis thaliana — Stability of small ubiquitin-like modifier (SUMO) proteases OVERLY TOLERANT TO SALT1 and -2 modulates salicylic acid signalling and SUMO1/2 conjugation in Arabidopsis thaliana — Supplementary Data 

# Stability of small ubiquitin-like modifier (SUMO) proteases OVERLY TOLERANT TO SALT1 and -2 modulates salicylic acid signalling and SUMO1/2 conjugation in *Arabidopsis thaliana*

## Supplementary Data

Data files

- Supplementary Data - Supplementary Data
