# Supplementary material for: Stability of small ubiquitin-like modifier (SUMO) proteases OVERLY TOLERANT TO SALT1 and -2 modulates salicylic acid signalling and SUMO1/2 conjugation in Arabidopsis thaliana
Source: J Exp Bot. 2015 Oct 22;67(1):353–63. doi: 10.1093/jxb/erv468 (PMC4682439; doi:10.1093/jxb/erv468)
Supplement: Supplementary Data [file supp_erv468_Supplementary_data.pdf]

Supplementary data

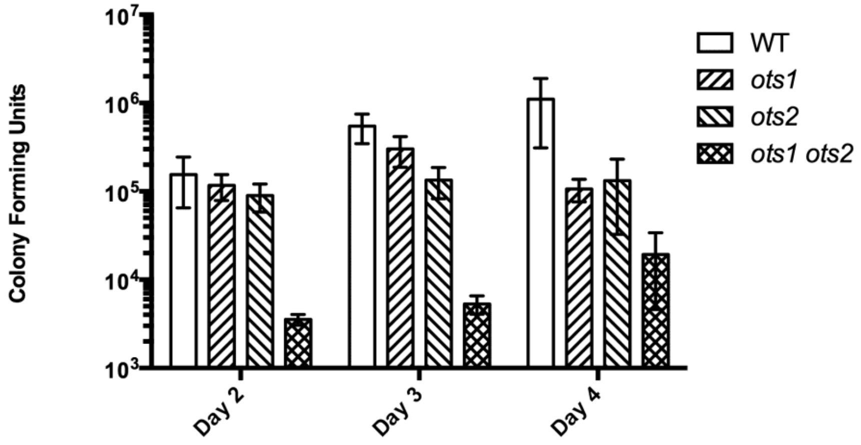

Supplemental Figure 1. Colony forming unit counts of *Pseudomonas syringae* pv *tomato* DC3000 from the leaves of 4 week old Arabidopsis plants; Wild-type (WT), single *ots1* and *ots2* mutants, and the double *ots1 ots2* mutants 2, 3 and 4 days post infiltration.

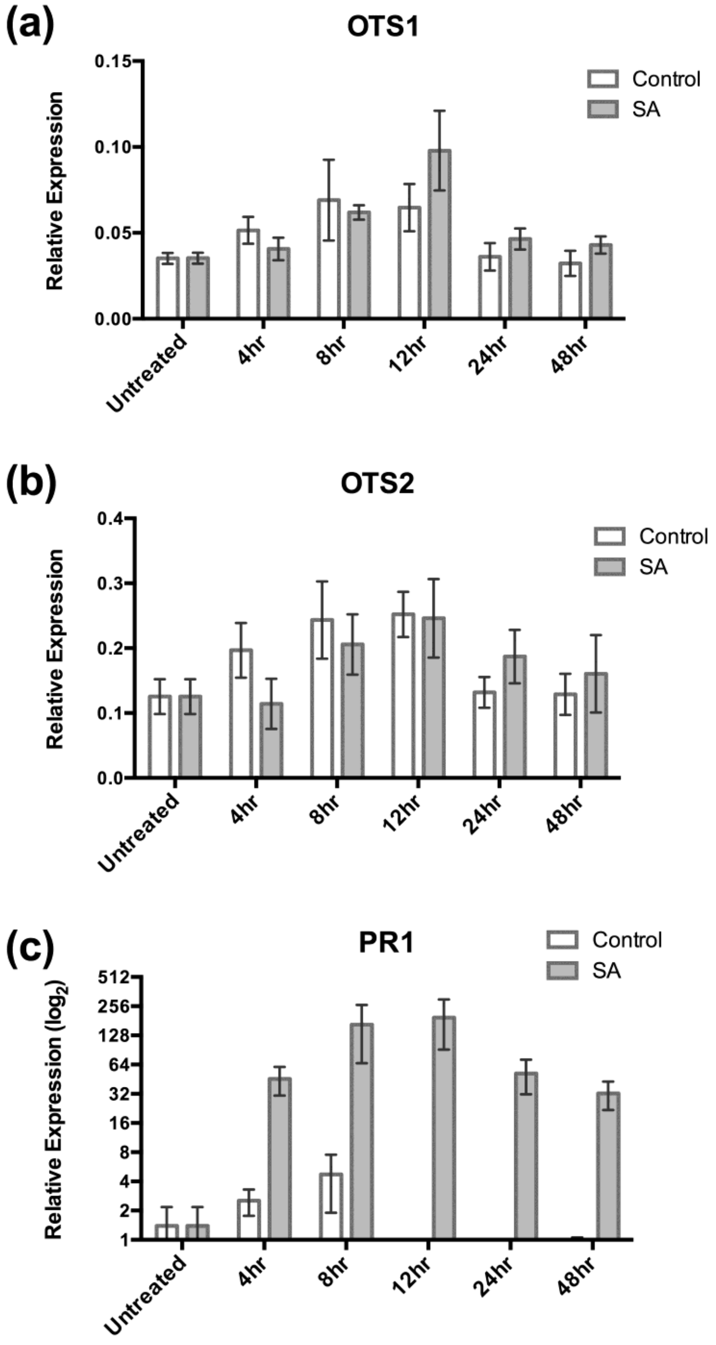

Supplemental Figure 2. *OTS1* and *OTS2* gene expression is unresponsive to SA treatment. Quantitative PCR gene expression analysis of **(A)** *OVERLY TOLERANT to SALT1* (*OTS1*), **(B)** *OTS2*, and **(C)** *PATHOGENESIS-RELATED1* (*PR1*) (normalized to *ACTIN7*), in 4 week old WT plants sprayed with salicylic acid or solvent control over a time-course of 48 hours. Error bars represent Standard Error of the Mean.

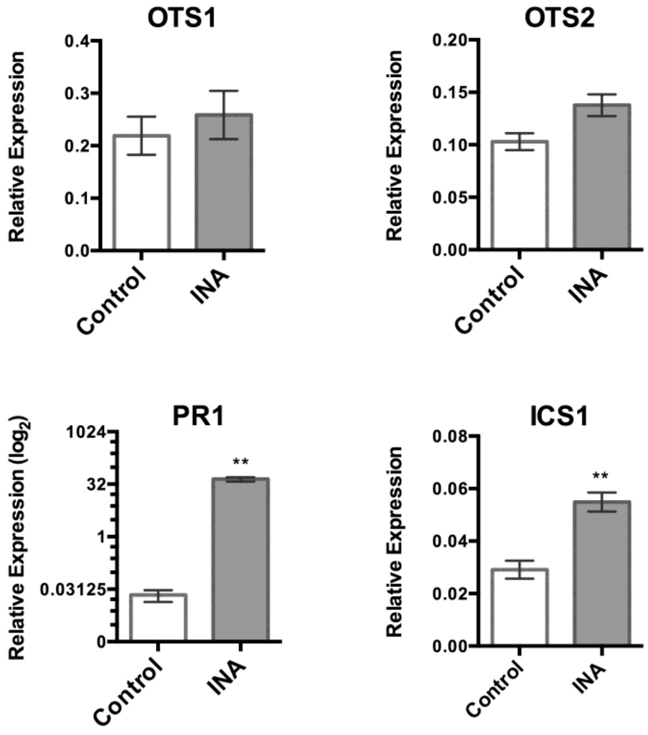

Supplemental Figure 3. *OTS1* and *OTS2* gene expression is unresponsive to INA treatment. Quantitative PCR gene expression analysis of *OVERLY TOLERANT to SALT1* (*OTS1*), *OTS2*, *PATHOGENESIS-RELATED1* (*PR1*) and *ISOCHORISMATE SYNTHASE1* (*ICS1*) (normalized to *ACTIN7*), in 10 day old WT seedlings grown in the presence of INA or solvent (Control). Error bars represent Standard Error of the Mean. ** *P* value 0.001-0.01 (unpaired Student's t-test).

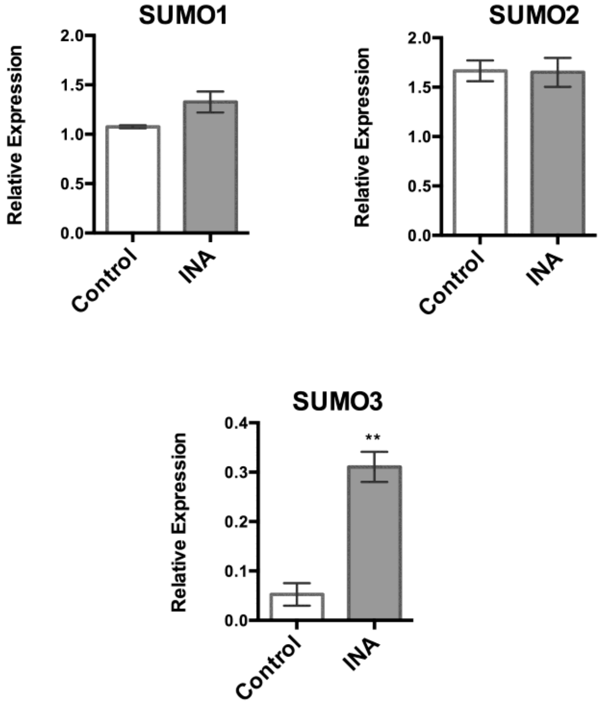

Supplemental Figure 4. *SUMO1* and *SUMO2* gene expression is unresponsive to INA treatment. Quantitative PCR gene expression analysis of *SUMO1*, *SUMO2* and *SUMO3* (normalized to *ACTIN7*) in 10 day old WT seedlings grown in the presence of INA or solvent (control). Error bars represent Standard Error of the Mean. ** *P* value 0.001-0.01 (unpaired Student's t-test).
